# Small bowel pigmentation

**Published:** 2016

**Authors:** Alaa Abdelwareth, Angus Molyneux, Ravi Madhotra, Sauid Ishaq, Kamran Rostami

**Affiliations:** 1 *Department of Gastroenterology, Milton Keynes University Hospital, United Kingdom*; 2 *Department of Pathology, Milton Keynes University Hospital, United Kingdom*; 3 *Dudley Group of Hospitals NHS Foundation Trust, United Kingdom*; 4 *Birmingham City University, Birmingham, United Kingdom*

**Keywords:** Pseudomelanosis duodeni, Small bowel pigmentation

## Question

A 73-year-old male, referred by his GP with dyspepsia unresponsive to proton pump inhibitors. His past medical history included COPD, ischemic heart disease, Iron deficiency anaemia (IDA) investigated with colonoscopy and a colonic polyp removed 2012. In the absence of a clear treatable condition behind his IDA, his low Iron was treated with Iron supplement Ferrous Sulphate. His current medications included esmoeprazole, ranitidine, atorvastain, losartan, inhalers, and ferrous sulphate. He was a heavy smoker, with a history of alcohol excess in the past. On Iron tablet his blood test revealed a normocytic anaemia with a Hb of 126, normal LFTs, U&Es, Amylase and CRP of 2.5. There was no history of blood transfusion and serum ferritin was 14 prior to the Iron therapy. He underwent an upper GI endoscopy in March 2016 and this showed a mild gastritis, moderate amount of food residue and multiple speckled areas of red pigmentation in the duodenum:

**Figure:**
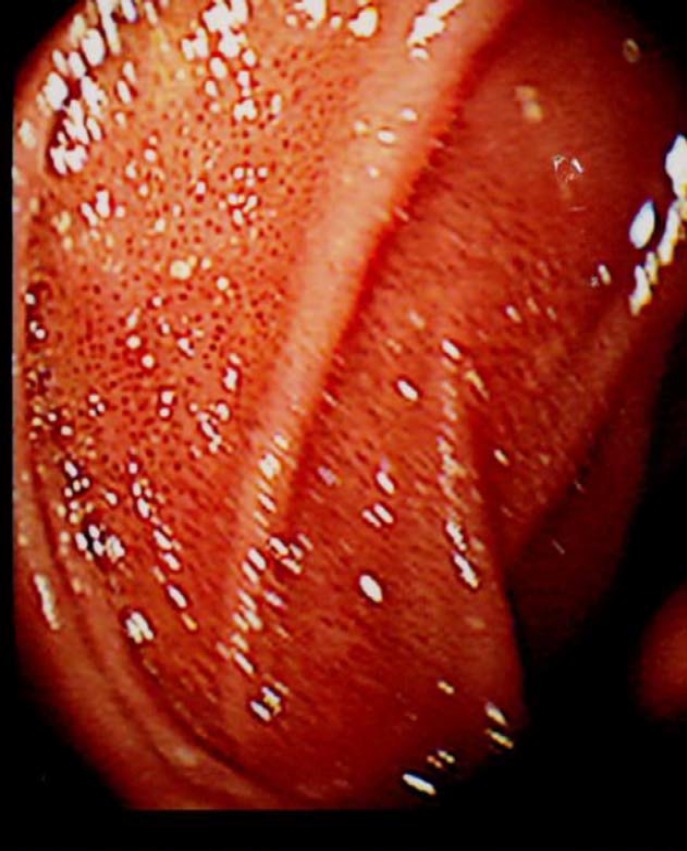


**What is your diagnosis and management?**

## Answer

**Figure 1:**
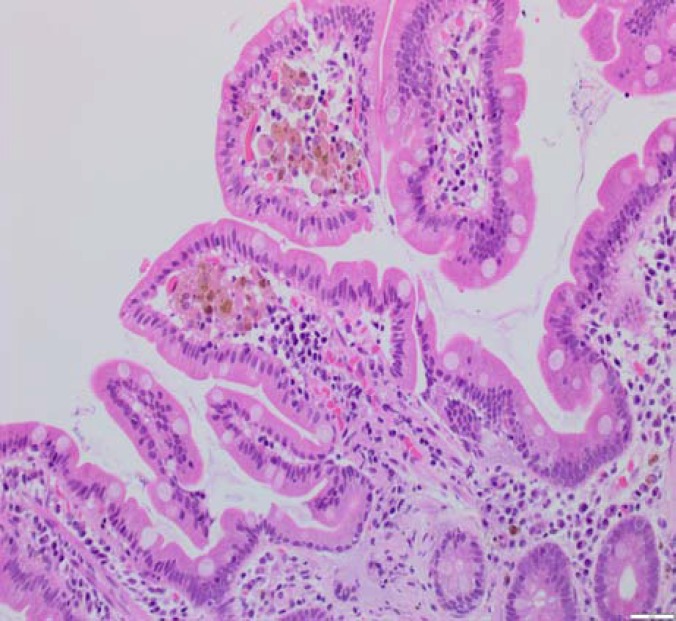
Hematoxylin and eosin stain. H&E stain shows golden-brown coloured coarse pigment granules in the tips of duodenal villi, mainly in macrophages

**Figure 2:**
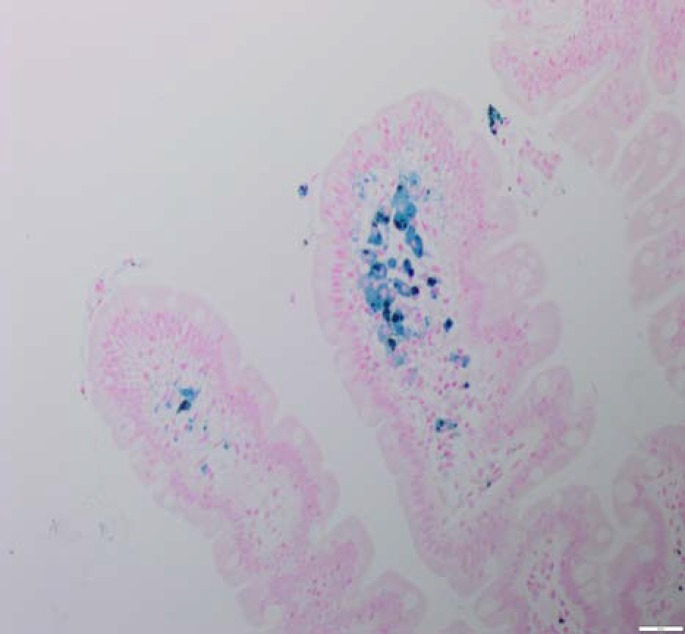
Perls’ stain for iron. Micrographs of duodenal mucosal biopsies (x200). Perls’ iron stain confirms that the pigment is haemosiderin

Biopsies of duodenal mucosa demonstrated hemosiderin deposits with positive iron staining, normal villous morphology with occasional small aggregates of haemosiderin-laden macrophages at the tips of the villi. Appearances were compatible with a diagnosis of duodenal haemosiderosis (See histology Figures 1-2). This is unusual but it is probably of no pathologic significance, although association with drug ingestion e.g., ferrous sulphate has been recorded. 

Hemosiderosis most often involves hepatic iron overload, but it has also been reported in patients on erythropoiesis stimulating agents and hemodialysis (1). This condition is also called Pseudomelanosis, however unlike the dark colour presentation described in other literature cases (2), the endoscopic feature in our case was consistent with red pigmentation most likely explained bytaking Iron supplements. The pigmentation usually normalises after removing the etiological factors.
